# Social Media and the Influence of Fake News on Global Health Interventions: Implications for a Study on Dengue in Brazil

**DOI:** 10.3390/ijerph20075299

**Published:** 2023-03-28

**Authors:** Marie-Catherine Gagnon-Dufresne, Mayana Azevedo Dantas, Kellyanne Abreu Silva, Jean Souza dos Anjos, Delano Pessoa Carneiro Barbosa, Rebeca Porto Rosa, William de Luca, Monica Zahreddine, Andrea Caprara, Valéry Ridde, Kate Zinszer

**Affiliations:** 1 Department of Social and Preventive Medicine, School of Public Health of the University of Montreal, Montreal, QC H3N 1X9, Canada; 2 Center for Public Health Research, Montreal, QC H3C 3J7, Canada; 3 Center for Health Sciences, Ceará State University, Fortaleza 60714-903, CE, Brazil; 4 Center for Humanities, Ceará State University, Fortaleza 60020-181, CE, Brazil; 5 Center for Applied Social Studies, Ceará State University, Fortaleza 60714-903, CE, Brazil; 6 Kuya—Ceará Design Center, Fortaleza 60010-010, CE, Brazil; 7 Population and Development Center, French National Research Institute for Sustainable Development, University of Paris, 75006 Paris, France

**Keywords:** fake news, social media, social acceptability, cluster randomized controlled trial, dengue, health interventions, global health, Brazil

## Abstract

Social media usage is growing globally, with an exponential increase in low- and middle-income countries. Social media changes the ways in which information-sharing occurs, intensifying the population’s exposure to misinformation, including fake news. This has important repercussions for global health. The spread of fake news can undermine the implementation of evidence-based interventions and weaken the credibility of scientific expertise. This is particularly worrisome in countries, such as Brazil, in a sociopolitical context characterized by a lack of popular trust in public institutions. In this project report, we describe our experience with the spread of fake news through the social media platform WhatsApp during the implementation of a cluster randomized controlled trial aimed at reducing dengue incidence in children in Fortaleza (Brazil). During initial visits to selected clusters, the research team was met with resistance. Then, soon after data collection started, fake news began circulating about the study. As a result, the research team developed strategies to dispel suspicion and further promote the study. However, the climate of violence and mistrust, coupled with the COVID-19 pandemic, forced the interruption of the study in 2019. The lessons learned from our experience in Fortaleza can be useful to other researchers and practitioners implementing large-scale interventions in this era of health-related misinformation.

## 1. Introduction

Social media has changed the ways in which communication and information-sharing occur [1]. As we have seen with the COVID-19 pandemic, the spread of misinformation (i.e., false or inaccurate information, regardless of intention), as well as disinformation (i.e., deliberately misleading information), and fake news (i.e., fabricated and fraudulent information presented as news by omitting or adding information to facts), can alter individual perceptions and shift behaviors with regard to the disease and public health measures [2,3,4]. Fake news has, however, been present long before COVID-19, rising with the global increase in social media presence and consumption of web-based information over the last few decades [4]. This has important repercussions for global health, as the extensive flow of information, both valid and invalid, allowed by social media, can make it difficult for the public to distinguish between fact and fiction [5]. The spread of misinformation can undermine the implementation of evidence-based interventions and weaken the credibility of governments, public health agencies, and scientific expertise [6]. In turn, this increased mistrust towards public institutions and authority figures can put population health at risk [5]. In this project report, we describe our experience with the spread of fake news through the social media platform WhatsApp in the context of a trial to prevent dengue fever in children in Fortaleza (Brazil). We contextualize this experience within the broader Brazilian sociopolitical context and the use of social media in the country, with a particular focus on WhatsApp. We then discuss the lessons learned from this experience for the implementation of other global health research and interventions.

### The Brazilian Context: WhatsApp, Activism, and Politics

Social media usage continues to grow globally, with an exponential increase in low- and middle-income countries due to technological advances allowing for people to form networks and participate in public debates on social, political, economic, environmental, cultural, and other issues [7,8]. In Brazil, a country marked by striking social inequalities, there is a history of social media being leveraged by the population for the purposes of political participation [7]. One example is notably the public demonstrations sparked by social media (notably through Facebook, YouTube, and Twitter) that have been organized in various Brazilian cities since 2013, condemning government corruption, economic disparities, as well as the lack of decent public healthcare, education, and public safety services [7,9]. 

Social media, including social networks such as WhatsApp is also emerging as a key news source for populations around the world [1,7]. WhatsApp is one of the primary sources of information for the Brazilian population [10]. Brazil represents the second-largest WhatsApp user base worldwide, with more than 146 million users [11]. WhatsApp is a free, multi-platform instant messaging application, in which information (i.e., calls, text messages, as well as video, audio, and image files) can be rapidly disseminated and shared between individuals or private groups of up to 256 members on the network, using internet data [1,12]. Because of its closed and encrypted architecture, WhatsApp affords a relative anonymity and security for users: its visibility is restricted for public authorities, it uses only phone numbers as identifiers, and allows for a limited number of members in a group [10,13]. While its primary purpose is social networking, the popularity of WhatsApp has also meant that it has been widely used as a channel for disseminating news and information, including fake news [11]. WhatsApp is particularly recognized as a fertile ground for groups interested in spreading disinformation, as it allows for the rapid sharing of messages, for which the sources are difficult to track [1,12]. These problems are exacerbated in contexts where internet access is unreliable, as users have a limited opportunity to fact-check the information received via WhatsApp [10].

During the 2018 presidential elections, it was noted that far-right candidates, such as former president Jair Bolsonaro used WhatsApp as the primary means of gaining political support [13]. Communication strategies based on an intense use of social media were employed for the micro-targeting of voters with specific characteristics and interests (i.e., religious, professional, regional, etc.) in WhatsApp chat groups [13]. WhatsApp was preferred over traditional media given its lack of legal provisions and monitoring mechanisms in the Brazilian electoral reform in comparison to other platforms, with regard to the distribution of electoral messages and their content [10]. The absence of regulations allowed for the sharing of hate-oriented, polarizing, conspiratorial, and biased content during the Brazilian electoral campaign [10,14]. These strategically constructed rumors were used to distort information on opposing parties and evoke emotional reactions from users, partly explaining Jair Bolsonaro’s election as president [10,13,14]. It is, however, worth noting that due to Bolsonaro’s use of WhatsApp to share propaganda during his campaign, the Brazilian Superior Electoral Tribunal has banned the sending of mass messages on social networks or instant messaging platforms for electoral purposes since 2019 [15].

It has been found that the spread of fake news via social networks such as WhatsApp during the COVID-19 pandemic contributed to discrediting scientific evidence and health institutions in Brazil and globally [16]. This could in part be attributed to the novelty of the pathogen and the continual changes in information and prevention measures, paralleled with poor risk communication, increased fear and frustration, and mistrust of the government and public health institutions [5,16]. In Brazil, Bolsonaro minimized the seriousness of the pandemic by spreading misinformation on symptoms, risks of transmission, protective measures, and potential treatments for COVID-19, and by instigating risky behaviors through traditional media (TV, radio, etc.) and social media platforms (Twitter, Instagram, Facebook, etc.) [17]. Bolsonaro mentioned that COVID-19 was a “little cold or flu” and that, “with [his] history as an athlete, if [he] were infected with the virus, [he] would have no reason to worry” [18]. He also posted on Twitter saying that “once again, hydroxychloroquine demonstrated its efficacy for people with COVID-19” [19]. Denialism, exemplified in Bolsonaro’s discourses on COVID-19, is considered a form of disinformation and can be defined as the denial of facts (e.g., historical, scientific) in an attempt to make people who are unaware of scientific data disbelieve evidence on a certain topic [20].

Similar to what was seen during his political campaign, the discrediting of scientific evidence, democratic institutions, and the press on social media by Bolsonaro during the COVID-19 pandemic has been coined digital populism [17,21]. Bolsonaro employed a divisive rhetoric of ‘us’ (including himself, his government, and the Brazilian population) versus a ‘corrupt elite’ (including traditional media and the judiciary) responsible for conspiring and benefitting from the pandemic [18,21,22]. For example, at the beginning of the pandemic, Bolsonaro’s narrative on Twitter portrayed his government as a provider responsible for meeting the needs of the people (economic aid, funds release, low taxes, etc.) [23]. He used two strategies to establish the legitimacy of his political choices during COVID-19. On the one hand, Bolsonaro constructed a narrative around his government as working to address people’s desires to return to work and put food on the table, which was in direct opposition to lockdown measures and economic shutdown [23]. On the other hand, he portrayed mainstream media, the judiciary, and other political parties as obstacles to be overcome. He represented them as corrupt, liars, criminals, and opportunists who were spreading fear, causing chaos, and ignoring his government’s decisions [23]. To justify his prioritization of the economy over public health, Bolsonaro therefore leveraged social media to create a COVID-19 imagery opposing the *united*, *strong*, and *working* people of Brazil to all those with decision-making power that would threaten the people’s ability to resume their normal lives [18,23].

Bolsonaro’s stance towards COVID-19 (including his refusal to follow the World Health Organization’s guidelines, his lack of support for social distancing and other protective measures, and his advocacy for hydroxychloroquine as treatment for COVID-19) led to unprecedented pressure on the country’s Ministry of Health [22,24]. The polarization between the president and the Ministry of Health led to the firing and resignation of two ministers during the pandemic period (finally replaced with an army general with little medical experience), creating political instability and confusion about COVID-19 among the population [22,24,25]. This is particularly worrisome in countries such as Brazil where the sociopolitical context is already characterized by a generalized lack of trust in public institutions and authority figures due to a long history of corruption [10].

It is worth emphasizing that in Brazil, there is little popular understanding of how corruption works, a situation which has been leveraged by far-right political movements to spread misinformation, blaming past progressive governments for poor accountability in public spending [26]. This has allowed for the formation of a political oligarchy in Brazil since 2012—involving economically powerful individuals and businesses, traditional media, and far-right political actors—resulting in private resources funding electoral campaigns, public funds being allocated following private interests, and in the renunciation by the press to scrutinize public spending [26]. 

Not only has this exacerbated mistrust in public institutions, but it has been identified as a direct threat to democracy in Brazil [26]. It has also been found that Jair Bolsonaro formed an office within his government, which has been called the “office of hate”, spreading fake news systematically and defaming political opposition on social media [27]. Considering the impacts that misinformation can have on public behavior, political outcomes and popular trust in government institutions, the general culture around the open and participatory use of social media like WhatsApp for civic engagement in Brazil raises concerns for global health [14]. In such a climate of mistrust, misinformation could potentially jeopardize the social acceptability of initiatives supported by governments and other public entities, such as those put in place to improve population health.

## 2. Materials and Methods 

The COESA study was a pragmatic cluster randomized controlled trial, implemented in 2019 and conducted by a partnership of Canadian and Fortaleza researchers, aimed at reducing dengue incidence in children aged 3–9 years old using a participatory mobilization approach in Fortaleza, in the northeastern Brazilian state of Ceará [28]. Dengue, transmitted by the Aedes mosquito, is the fastest-spreading vector-borne viral disease worldwide. It is endemic in Brazil, with over 2.2 million cases reported in 2019, with a national incidence of 1078 cases per 100,000 inhabitants [29,30]. Fortaleza is particularly burdened by dengue, with children under the age of 15 representing most of the cases [28,31]. Its tropical climate, high population density, rapid urbanization, and inadequate sanitation conditions make the population of Fortaleza particularly vulnerable to dengue [28,32]. With high poverty rates and income inequalities, Fortaleza is also one of the most violent cities in the country and worldwide due to drug trafficking and related gang violence [32]. In the last decades, violent crime rates have escalated in Fortaleza, as gangs have attempted to kill members of rival gangs and control the local drug market [33]. Studies conducted in Fortaleza have shown that violent crimes perpetrated by gangs have clustered in neighborhoods with high income inequalities, high population density, low literacy levels, and limited access to public infrastructure [32,33]. These socioeconomically deprived neighborhoods also are particularly at risk of dengue [34].

As there exists no treatment for dengue and vaccines are not yet widely available, preventing or reducing dengue transmission largely depends on controlling Aedes mosquitoes and limiting human-mosquito contact [28]. The COESA study was modelled upon the successful Camino Verde trial conducted in Nicaragua and Mexico, which demonstrated the effectiveness of community mobilization for reducing dengue incidence in children [35]. The community mobilization approach, called Socializing Evidence for Participatory Action (SEPA), is a participatory approach to health promotion, in which communities develop their own solutions to reduce dengue incidence in their community [36]. Community mobilization activities developed and implemented by community members in Nicaragua and Mexico notably included education initiatives in schools, the closing of water tanks to prevent mosquitoes from laying egg, and biological control using larvivorous fish [37]. The different types of data collected in the study involved household surveys, entomological surveys, and finger-prick blood samples from the participating children [28]. 

Following Camino Verde’s success, the COESA study was launched in Fortaleza in 2019. The COESA research team was formed of Canadian and Brazilian researchers. Principal and co-investigators were trained in a diversity of disciplines (i.e., public health, epidemiology, medical anthropology, entomology, participatory research) with experience working on arboviruses, including dengue. Brazilian researchers had extensive field experience in Fortaleza with existing ties to community leaders, community health workers, and entomological agents in the neighborhoods targeted for the study. Their combined experienced, along with that of the principal investigator of the Camino Verde trial, directly informed the planning of the COESA study, a two-year process prior to data collection. The research team consulted various local stakeholders during the planning phase of the study, including researchers from other local institutions, teams within the municipality of Fortaleza, and the unions for entomological and community health workers.

When the selection of clusters began, the objective was to have 34 clusters in each arm with 86 children per cluster, for a total of 5849 children [28]. Between June and August 2019, the cluster selection started through field visits by the local research team to establish contact with the clusters and document the context. At this stage, clusters deemed unsafe for study personnel were excluded. The first participants were recruited in November 2019, followed by baseline data collection comprising of a finger-prick blood collection, as well as a child and household survey. 

### Ethics

The study was conducted in accordance with the Declaration of Helsinki. It was approved by the Health Research Ethics Committee of the University of Montreal (reference number 18-141) and the Research Ethics Committee of Ceará State University (reference number 3.083.892). All participants involved gave their written informed consent to participate in the study before their enrolment.

## 3. Results

### 3.1. Mistrust, Fake News, and Resulting Implications

From the start of the study, the research team met multiple challenges. First, the aggressive and divisive rhetoric employed by former president Bolsonaro since his election in 2018 seemed to have created a generalized climate of distrust in the country, including towards science and scientific institutions that have repeatedly been discredited by the government [38,39]. Second, the escalation of gang violence in Fortaleza since the beginning of 2019 exacerbated the already tense sociopolitical context, igniting fear among the population. Organized in retaliation to the Government’s decision to separate gang factions in Brazil’s prisons, gangs in Fortaleza began carrying out attacks against government vehicles and buildings, and on main roads around the city [40]. This context of mistrust and violence had important repercussions on the implementation of COESA. 

During the first field visits and baseline data collection, the presence of study personnel (field teams formed of undergraduate and graduate students from Fortaleza) in many of the clusters, some of which were controlled by gang factions, was met with suspicion and resistance. To tackle this issue, the research team sought the collaboration of municipal workers specialized in infectious diseases and community health agents, in an attempt to improve the relations between the research team and community members. Yet, this collaboration with local workers was not very fruitful, as they appeared to be uninterested to mobilize and contribute to the study. We believe that the lack of financial compensation for their collaboration efforts was an important barrier, which was not possible to provide given budgetary constraints.

In January 2020, soon after the baseline data collection started, fake news began circulating about COESA and its personnel. Multiple messages were shared on WhatsApp, warning cluster residents about untrustworthy individuals pretending to be affiliated with a university (i.e., the research team) visiting households. These messages stressed that these individuals were collecting personal information on children, that they were refusing to show their material to community members, and that they were making children sick by injecting them with an unknown liquid after collecting blood, sending some of the children to the hospital because of the infection they were given. Here are some examples of the messages that were circulating in Fortaleza about the study:

*Hi, good morning! I am sending this zap to […] my list of contacts, because a very strange thing is happening […]. A team is passing by […] they are walking around with a blue coat and badges, saying that they are from a university […]. They come […] and ask how many people are in each house, how long they have lived there, these silly research questions. […] If there are children in the house, [they ask] how old they are. […] They [say they] need to take blood […] to know if they have already contracted dengue. […] I am not crazy [enough] to bring my children to the door for a person I don’t even know. They also don’t want to show their material. Woman, they deceive, brainwash people […]. The neighbor […] gave away his little two-year-old girl […]. They left here highly indignant, angry with me. […] If it is a contaminated syringe or some liquid even to kill the child… You stay tuned!*

*People, good afternoon! […] The story told in these [audio messages] is true! Two children who have been contaminated after blood collection have already arrived here at the [local hospital]. I don’t know what the diagnosis was. They all must be examined. One of them has already been hospitalized. I will call, now, to tell the father about this situation. […] Because two children have already arrived. When asked about the diagnosis, they said: “They took blood and then vomited”, and they are not doing well.*

While the origins of the fake news circulating about COESA are unknown, the local research team hypothesized on different potential sources. These included gang members in the clusters, who were displeased with the presence of the research team in their territory. Another hypothesis is that the communities themselves spread the fake news to protect their children, as they were worried that the research team was using blood samples for purposes other than the research. These messages started not only circulating in the communities targeted by the study, but also in surrounding neighborhoods and municipalities. 

### 3.2. Strategies Developed to Counter Fake News

To address the fake news situation, the research team intensified their communication activities, working with local media channels and community health clinics to further promote and dispel any suspicions about COESA. The team also sought support from local decision makers, and organized meetings and presentations with various groups and local institutions. As part of these efforts, a community liaison team was created. The team met with communication consultants from the municipality of Fortaleza and the Ceará state government. Presentations were then organized in churches, factories, and community radio stations in the different clusters to provide more information about COESA to the local populations.

Despite these efforts, strategies to counter the misinformation were insufficient and the messages continued to circulate, resulting in increasingly aggressive reactions to study personnel and near consistent refusals to participate from community members. In February 2020, coinciding with the arrival of the COVID-19 pandemic, Fortaleza experienced a police riot: police officers illegally organized riots and protests to demand better working conditions, further escalating violence in the city [41]. The study was then stopped because of these events, along with the pandemic, as the context of violence coupled with the risk of COVID-19 exposure made it too unsafe to continue with the study.

## 4. Discussion: Lessons for Global Health

Our experience with the spread of fake news via WhatsApp during the COESA study in Brazil stresses the importance of reflecting on the broader implications of contexts with intensive social media use and high levels of circulation of health-related misinformation. Countries in which there is a high use of social media, low health literacy, low government trust, and limited access to accredited sources of information are particularly susceptible to the spread of health-related misinformation [5,10,14]. There have been multiple reports of the influence of social media on the ways in which interventions are implemented for a variety of health issues, including infectious diseases such as Ebola, Zika, and COVID-19 [5,42]. 

A literature review highlighted that misinformation was common on all aspects (i.e., prevention, treatments, risk factors, transmission modes, complications, vaccines) of large-scale infectious disease outbreaks since 2000 [5]. For instance, the spread of fake news and conspiracy theories on vaccines for COVID-19 in sub-Saharan Africa increased vaccine hesitancy, causing confusion and fear among the general population, derailing governments’ efforts at controlling the spread of the virus in their territories [43,44]. Yet, the conflicting results of studies assessing the association between sociodemographic characteristics (i.e., age, gender, education level) and the vulnerability to believe or share misinformation in the context of a public health crisis suggest that people of all backgrounds are susceptible to misinformation [4,5,45,46].

The increased spread of misinformation about health calls out for the development of guidelines to support researchers and practitioners in preventing and addressing issues related to social media usage and public mistrust. Existing recommendations for refuting misleading information on pressing public and global health issues include developing a clear messaging strategy and providing appropriate sources of information; improving risk communication by addressing public concerns and needs; engaging community members and working with community leaders; leveraging information technology and partnering with social media platforms to encourage fact checking; and using social media to reach the population [42,45,47,48,49]. 

With hindsight, there are a few crucial steps that we should have taken prior to the implementation of the COESA study to lessen the risk of mistrust from the population. We should have conducted a thorough assessment of social media use in Fortaleza to better understand its role in disseminating and accessing health information, which should also have included looking for accounts of similar situations experienced by local researchers and authorities, to learn from the solutions they developed. This would have provided us with the blueprint to build and pilot test a robust communication strategy prior to beginning the study, to anticipate potential repercussions.

We should also have included community leaders and other community members in the study prior to baseline data collection. The initial problems with resistance to data collection were unanticipated, despite having several local researchers as part of the investigation team. The original plan was to include these stakeholders once the baseline collection was finished, in order to present the baseline results, obtain their feedback, and encourage their support and potential participation in the community mobilization activities. Having their input and support prior to the baseline would have likely helped with the acceptability of the study and would have potentially reduced the reach of the fake news. Table 1 presents a summarized list of recommendations for global health researchers and practitioners, developed from the lessons learned from the implementation of the COESA study in Brazil.

## 5. Conclusions

Our experience with COESA speaks to the growing importance of social media and its repercussions for how we implement interventions in global health. We hope that it encourages others to better understand the sociopolitical contexts and the role of social media for information sharing in the countries in which projects are being implemented. Considering the speed at which information is shared on social media, we also stress the necessity to work closely with local communities to understand the importance of social media within their networks, to ensure that projects are socially acceptable, and to identify potential dangers or challenges in the study or its messaging.

## Figures and Tables

**Table 1:** Recommendations for global health researchers and practitioners.

**Recommendations** Conduct a thorough assessment of social media usage in the context where research is to be implemented; Develop a robust communication strategy using local communication channels to promote the study prior to its implementation; Involve local decision makers, community leaders, and community members in information dissemination from the beginning of the study to improve its social acceptability.

## Data Availability

Not applicable.
